# Function Identification of Bovine *ACSF3* Gene and Its Association With Lipid Metabolism Traits in Beef Cattle

**DOI:** 10.3389/fvets.2021.766765

**Published:** 2022-01-06

**Authors:** Wei He, Xibi Fang, Xin Lu, Yue Liu, Guanghui Li, Zhihui Zhao, Junya Li, Runjun Yang

**Affiliations:** ^1^College of Animal Science, Jilin University, Changchun, China; ^2^College of Coastal Agricultural Sciences, Guangdong Ocean University, Zhanjiang, China; ^3^Institute of Animal Sciences, Chinese Academy of Agricultural Sciences, Beijing, China

**Keywords:** Acyl-CoA synthetase family member 3, Chinese Simmental cattle, fat deposition traits, single nucleotide polymorphism, triglyceride (TG)

## Abstract

Acyl-CoA synthetase family member 3 *(ACSF3)* carries out the first step of mitochondrial fatty acid synthesis II, which is the linkage of malonate and, to a lesser extent, methylmalonate onto CoA. Malonyl-coenzyme A (malonyl-CoA) is a central metabolite in mammalian fatty acid biochemistry that is generated and utilized in the cytoplasm. In this research, we verified the relationship between expression of the *ACSF3* and the production of triglycerides (TGs) at the cellular level by silencing and over-expressing *ACSF3*. Subsequently, through Sanger sequencing, five polymorphisms were found in the functional domain of the bovine *ACSF3*, and the relationship between *ACSF3* polymorphism and the economic traits and fatty acid composition of Chinese Simmental cattle was analyzed by a means of variance analysis and multiple comparison. The results illustrated that the expression of *ACSF3* promoted triglyceride synthesis in bovine mammary epithelial cells and bovine fetal fibroblast cells. Further association analysis also indicated that individuals with the AG genotype (g.14211090 G > A) of *ACSF3* were significantly associated with the fatty acid composition of intramuscular fat (higher content of linoleic acid, α-linolenic acid, and arachidonic acid), and that CTCAG haplotype individuals were significantly related to the fatty acid composition of intramuscular fat (higher linoleic acid content). Individuals with the AA genotypes of g.14211055 A > G and g.14211090 G > A were substantially associated with a larger eye muscle area in the Chinese Simmental cattle population. *ACSF3* played a pivotal role in the regulation of cellular triacylglycerol and long-chain polyunsaturated fatty acid levels, and polymorphism could serve as a useful molecular marker for future marker-assisted selection in the breeding of intramuscular fat deposition traits in beef cattle.

## Introduction

As there are large numbers of Chinese Simmental cattle breeding groups, verifying the gene function of this breed's high-quality traits is an area in need of urgent research. The Chinese Simmental has become a large-scale dairy and meat breed in China since it was successfully selected and bred in 2001. In recent years, with the maturity of sequencing technology, new association studies have been conducted to assess whether genetic polymorphisms affect the economic traits and fatty acid composition in cattle (1, 2). Molecular marker information can be used as a useful tool for evaluating breeding value and selecting animal carcass and meat traits.

Fatty acid synthesis occurs through two pathways, one of which takes place in cellular structures called mitochondria. Mitochondria convert the energy from food into a form that cells can use, and fatty acid synthesis in these structures is essential for their proper functioning. The ACSF3 enzyme is found only in the mitochondria and is involved in mitochondrial fatty acid synthesis. The *ACSF3* provides instructions for making an enzyme involved in the formation (synthesis) of fatty acids, which are the building blocks used to make fats (lipids) (3). The *ACSF3* enzyme performs a chemical reaction that converts malonic acid to malonyl-CoA, which is the first step of fatty acid synthesis (4). Based on this activity, the enzyme is classified as a malonyl-CoA synthetase (5). The ACSF3 enzyme also converts methylmalonic acid to methylmalonyl-CoA, making it a methylmalonyl-CoA synthetase as well (6). Studies have shown that the single nucleotide polymorphism of *ACSF3* may be related to meat quality traits, such as backfat thickness (7, 8). The bovine *ACSF3* gene is located on bovine chromosome 18:14,209,800–14,248,433 and has 10 exons and 9 introns. The *ACSF3* mRNA sequence contains a 1,761 bp coding region (CDS) and a 276 bp untranslated region. The open reading frame (ORF) encodes 586 amino acids and has a 94 and 84% homology with the coding regions of sheep and swine, respectively. The transcriptome analysis results of our previous study showed that *ACSF3* was a candidate gene related to bovine fat deposits. However, the *ACSF3* gene has been rarely studied in bovine lipid metabolism.

This study aims to reveal the regulatory effect of *ACSF3* on adipogenesis by intracellular gene RNA interference (RNAi) and gene over-expression. Moreover, we investigate the polymorphisms of the bovine *ACSF3* gene functional domain to explore the relationship between genotypes and fat deposition, and the fatty acid composition traits of Chinese Simmental cattle.

## Materials and Methods

### Ethics Statements

All the animal experiments in the present study strictly complied with the relevant regulations regarding the care and use of experimental animals issued by the Animal Protection and Use Committee of Jilin University [permit no. SYXK(Ji) pzpx20181227083].

### Experimental Materials

The BMECs (bovine mammary epithelial cells) in this study were purified and cultured according to the previous work carried out by the Laboratory of Bovine Genetic Resources and Functional Genomics (9). Briefly, bovine mammary tissues were cut into 1 mm^3^ nubbles and washed again with PBS solution until the tissue was clean. The smaller pieces of tissue were transferred onto cell culture dishes. A basal media was prepared in advance: DMEM/F12 with 10% fetal bovine serum, 1% penicillin and streptomycin (Hyclone, Logan, UT, USA), and 1% epithelial growth factor. Next, the cell culture dishes with the tissue were incubated at 37°C in a 5% CO_2_ incubator. After 6 h, 5 mL of basal media were added to each dish, ensuring that the tissue would not float and separate from the bottom of the culture dish. The basal media was replaced with fresh media every 48 h until the culture dish was full of cells. The cells were detached with 0.25% trypsin-0.02%EDTA (Hyclone, Logan, UT, USA) and transferred to new culture dishes that were used to remove the fibroblasts. Subsequently, pure mammary epithelial cells were isolated after 3–5 passages.

The BFFs (bovine fetal fibroblast cells) were isolated and preserved by the Genetic Breeding and Reproduction Laboratory of Jilin University. Briefly, the BFFs were obtained by isolating fetal bovine ear tip tissue using a tissue block apposition method similar to that described above.

This study involved 135 Chinese Simmental cattle (28-month-old bulls) from a Baolongshan cattle farm in Inner Mongolia. These cattle were randomly selected from the offspring of a Simmental cattle population with ~2,000 cows and 25 bulls. Blood samples (10 mL each) were collected from jugular vein with anticoagulant (Acid citrate dextrose, ACD) and stored in −70°C. DNA was extracted from 1 mL whole blood with the DNA extraction kit (Tiangen, Beijing, China) according to the manufacturer's protocol.

### Trait Analysis

In this study, 36 traits were measured and 14 fatty acid compositions of the *Longissimus dorsi* muscle were analyzed (10, 11).

Before the measurements of carcass and fat deposition traits, all the carcasses were stored in refrigerating chambers at the temperature between 0 and 4 °C for 24 h. All the measurements complied with the criterion GB/T17238-1998 cutting standard of fresh and chilled beef of China (China Standard Publish). Before slaughter, ultima live backfat thickness, body weight and *longissimus* muscle area (by ultrasound) were recorded. The weights of carcass, omental fat, mesenteric fat, kidney fat (stripping kidney fat and weighting) was recorded at the slaughter plant. And we also record fat coverage rate, marbling score, fat color score, muscle color score, rib eye area, and backfat thickness after slaughter. Weight of kidney fat was expressed as an absolute value and also as a percentage of hot carcass weight. Carcass yield was calculated as (hot carcass weight × 100)/final weight. Marbling was measured by video image analysis and expressed as the percentage of visible fat area over the total area of a steak (*Longissimus thoracis* muscle taken between the 9 th and 10 th vertebrae from the right side of the carcass). Moreover, the carcass length (measuring the shortest length between the bun midpoint to the sciatic trailing edge), carcass chest depth (measuring the shortest distance of the test cattle between bun posterior border to pectoral by using a ruler), hind leg circumference (measuring the surrounded degree in the junction of femur tibia and fibula), hind leg width (measuring the horizontal width since the end of the medial sag to thigh front), thigh meat thickness (the vertical distance from the surface to the midpoint of the autologous femoral body) and other carcass traits were recorded at the slaughter plant.

Briefly, intramuscular fat was obtained from the *Longissimus dorsi* muscle. The analysis was performed in accordance with the ISO 5,509 (2,000) norms, and AOAC procedures (Association of Official Analytical Chemists), and expressed as g/100 g of fresh tissue. The intramuscular fat was collected in a glass methylation tube, mixed with 4.0 mL of MeOH, 2.0 mL of chloroform (capillary GC; Sigma-Aldrich, MO, USA), and 1.5 mL of ddH_2_O, and placed in a rotating platform shaker for 10 s, then left at room temperature for 15 min. Chloroform and ddH_2_O were added to the wells and mixed again, and left at room temperature for 5 min. The mixture was centrifuged at 1,500 g/min for 30 min, then cooled to 16°C, the upper layer removed, and dried under nitrogen. Next, 1.5 mL of 14% BF_3_/MeOH reagent were added and mixed vigorously. The mixture was heated at 90°C for 30 min, cooled to room temperature, and 4.0 mL of hexane was added, then methyl esters were extracted in the hexane phase following the addition of 1.5 mL of ddH_2_O. The samples were centrifuged for 1 min, and then the upper hexane layer was removed and concentrated under nitrogen. Fatty acid methyl esters were analyzed by gas chromatography using a fully automated HP5890 system (Agilent, Santa Clara, CA, USA) equipped with a flame-ionization detector. An SP-2,560 column (Supelco, PA, USA) 100 m × 0.25 mm × 0.20 μm was used for gas chromatographic detection. The detection procedure was in an oven at 140°C for 5 min, 10°C/min to 220°C, and held for 50 min. An injector/detector was used at 260°C with helium as a carrier gas at about 5 psi. The identification of components was carried out by a comparison of the retention times with those of authentic standards (Sigma-Aldrich, MO, USA).

### Primer Design

Primer Premier 6 software (PREMIER Biosoft, San Francisco, CA, USA) was utilized to design the *ACSF3* gene coding sequence primers and the SNP primers, based on the bovine *ACSF3* gene's existing published sequences (ENSBTAG00000015968).

### Construction of pGPU6/GFP/NEO-sh*ACSF3* Vector and pBI-CMV3-*ACSF3* Vector

The constructed RNAi-vector pGPU6/GFP/NEO-sh*ACSF3* with a size of 5276 bp and containing the shRNA sequence of bovine *ACSF3* gene. The enhanced green fluorescent protein (EGFP) gene was used for transient expression in cells, and kanamycin was used as a selection marker for prokaryotic cell (DH5α) amplification (**Figures 2A-II**). The shRNA target sequences of bovine *ACSF3* mRNA were screened and designed by BLOCK-iT™ RNAi Designer (https://rnaidesigner.thermofisher.com/rnaiexpress/, Table 1). The shRNA sequence was cloned into a pGPU6/GFP/Neo vector (GenePharma Corporation, Shanghai, China) using the *Bbs*I and *BamH*I (New England Biolabs, MA, USA) restriction enzyme digestion method. The eukaryotic expression vector pGPU6/GFP/Neo was double digested with *Bbs*I and *BamH*I in a 10 μL reaction system: *Bbs*I 0.75 μL, *BamH*I 0.75 μL, NEBuffer 2.1™ 1 μL, pGPU6/GFP/Neo plasmid 2 μL, Nuclease-free water 4.5 μL, and incubated at 37°C 5 h. Recovered and ligated by T4 DNA Ligase (Takara, Dalian, China): T4 DNA Ligase Buffer 2 μL, T4 DNA Ligase 1 μL, pGPU6/GFP/NEO vector 1 μL, shRNA fragment 1 μL, Nuclease-free water 15 μL and incubated at 4°C overnight. The products were transformed into competent *E. coli* (DH5α) cells (Tiangen, Beijing, China), and then prepared the pGPU6/GFP/NEO-sh*ACSF3* plasmid. The kanamycin-positive clones were picked out and transferred to 200 mL of liquid Luria-Bertani (LB) medium and shaken overnight at 37°C. The pGPU6/GFP/NEO-sh*ACSF3* plasmids were extracted using the EndoFree Maxi Plasmid Kit (Tiangen, Beijing, China) according to the manufacturer's protocol. Briefly, the bacterial broth was centrifuged to obtain the precipitate, fully lysed and removed the endotoxin, and rinsed with enzyme-free water to obtain the plasmid. The concentration and purity of the plasmids were assessed using the agarose gel and spectrophotometer (Nanodrop 2,000, Thermo Scientific, Waltham, MA, USA).

**Table 1:** The primer sequences.

**Primer**	**Forward sequences (5^′^-3^′^)**	**Reverse sequences (5^′^-3^′^)**	**Target sequences**
SNPs primer for *ACSF3*	TGTGACCTCAGTGCCTTCTT	CCTACATTTCTGGGTGCTTCC	
shRNA of bovine *ACSF3*	AGAGGTATAAGGACCTCTACTTGCGTTCAAGAGACGCAAGTAGAGGTCCTTATACTTTTTTG	GATCCAAAAAAGTATAAGGACCTCTACTTGCGTCTCTTGAACGCAAGTAGAGGTCCTTATAC	GTATAAGGACCTCTACTTGCG
q-PCR primer of *ACSF3*	GTGGCTGTGATTGGAGTT	TCTCGCTTGTTGACCTTC	
*ACACA*	GAAGGAGCGAGAGGAGTT	GTAGAAGAAGGTGCGTGAA	
*ACACB*	CAAGATTGCCTCCACCAT	CCTCCTGTAGACTGTGTTC	
*MCAT*	AAGGCGATTGATGTCAAGA	CTTCCTCCTCTCGTATATGG	
*FASN*	CACGAACAACAGCCTCTT	GCCTCCAGCACTCTACTA	
*CEBPα*	TGGACAAGAACAGCAACGAGT	GGTCATTGTCACTGGTCAGCT	
*β-actin*	AGAGCAAGAGAGGCATCC	TCGTTGTAGAAGGTGTGGT	

The constructed overexpression vector pBI-CMV3-*ACSF3*, which is 5,539 bp in size and contains the sequence of the CDS region of bovine *ACSF3* gene. The reef coral *Zoanthus sp*. green fluorescent protein (ZsGreen) gene was used for transient expression in cells and ampicillin was used as a selection marker (**Figures 2A-IV**). The CDS region of *ACSF3* synthesized by Sangon Biotech (Shanghai, China) was ligased into a pBI-CMV3 vector (Clontech Laboratories, Mountain View, CA, USA) using the *Mlu*I and *Hind*III (New England Biolabs, MA, USA) enzyme digestion method. Briefly, using the method similar to that described above, the resistance was replaced with ampicillin. The ampicillin-positive clones were picked out and transferred to 200 mL of liquid Luria-Bertani medium and shaken overnight at 37°C. The pBI-CMV3-*ACSF3* plasmid was extracted using the EndoFree Maxi Plasmid Kit (Tiangen, Beijing, China) according to the manufacturer's protocol.

### The Culture and Transfection of BMECs and BFFs

The BMECs and BFFs were proliferated for 24 h in six-well plates (Jet Bio-Filtration Co., Ltd, Guangzhou, China), and the final concentration of cells was 1.2 × 10^6^ cells per well. Each well was supplemented with 2 mL of growth medium containing DMEM/F12 (Corning, NY, USA) and 10% fetal bovine serum (FBS; Tian Hang, Zhejiang, China), then incubated at 37°C in a 5% CO_2_ incubator (Thermo Fisher Scientific, Massachusetts, USA).

In this study, the cells transfected with pGPU6/GFP/NEO-sh*ACSF3* vector were used as RNA interference group, and cells transfected with pGPU6/GFP/NEO vector were used as the control. While the cells transfected with pBI-CMV3-*ACSF3* vector were used as the *ACSF3* gene over-expression group, cells transfected with pBI-CMV3 vector were used as the control. After referring to similar experimental methods in published articles, this experiment adopted a transient transfection method, using the cells 48 h after the vector was transfected into the cells for subsequent experiments (12, 13).

For transfection, each vector DNA (3.0 μg) and 7.5 μL of FuGENE HD transfection reagent (Promega, Madison, Wisconsin, USA) were diluted in 150 μL of DMEM/F12 media and mixed lightly. The mixture was incubated at room temperature for 15 min and then added to the pores of each six-well plate. The cell culture medium was replaced after 6 h of transfection. After 36 h of transfection, green fluorescent protein (GFP) expression was detected with a fluorescence microscope (NikonTE2000, Tokyo, Japan). The efficiency of transfection was also determined by calculating the ratio of cells positive for green fluorescent protein expression to the total number of cells. The experiment was repeated three times.

### Analysis of mRNA Levels of *ACSF3* in Transfected BMECs

The transfected cells were collected for analysis of the mRNA expression levels. RNAiso Plus reagent (Takara, Dalian, China) was used to extract the total RNA from cultured cells for reverse transcription. cDNA was synthesized using a reverse transcription kit (TransGen Biotech, Beijing, China), 1 μg of total RNA, 4 μL of 5 × EasyScript^®^ All-in-One SuperMix for qRT-PCR, and 1 μL of gDNA remover, and RNA-free water was added to make the total volume of 20 μL. The cDNA synthesis conditions were: incubate at 42°C for 15 min, and at 85°C for 5 s. Quantitative real-time PCR (qRT-PCR) was followed through with SYBR Green Real-Time PCR Master Mix (Takara, Dalian, China) utilizing the specific primers shown in Table 1. qRT-PCR was performed in a 10 μL reaction with 5 μmoles in 0.5 μL of forward primer, 5 μmoles in 0.5 μL of reverse primer, 5 μL of SYBR Green Real-Time PCR Master Mix (Roche, Basel, Switzerland), 1 μL of cDNA, and 3 μL of ddH_2_O, using the following procedure: 95°C for 30 s and 45 cycles of 95°C for 5 s, and 60°C for 30 s in a PCRmax (Eco, Staffordshire, UK). Experiments were repeated three times. Three technical replicates were analyzed for each sample and β*-actin* was used as an internal standard to normalize the mRNA expression level using the 2^−Δ*ΔCT*^ method.

### Determination of Triglyceride Content in Cells of *ACSF3* Interference and Overexpression

In BMECs after interference or the overexpression of *ACSF3*, the detection of TGs was performed with a tissue and cell TG assay kit (Applygen Technologies Inc., Beijing, China) according to the manufacturer's protocol. Meanwhile, *ACSF3* was over-expressed in the BFFs and the TG content was examined. The optical density of each sample was determined using a microplate reader (Yong Chuang SM600, Shanghai, China). The experiment was repeated three times and three technical replicates were performed for each sample. The data regarding the triglyceride in the cells were adjusted based on the quantity of the protein, and the cellular content of triglyceride was corrected for the protein concentration of each μg.

### Polymorphic Loci Detection in *ACSF3* Gene and Genotyping

We used a 20 μL system for PCR amplification, combining 10 pmol/μL of each primer, 140 ng of bovine genomic DNA, and 10 μL of green Taq mix, according to the manufacturer's protocol (Vazyme, Nanjing, China). The PCR amplification conditions were as follows: incubation of the PCR mixture at 95°C for 5 min, 35 cycles of 95°C for 30 s, the annealing temperature of each fragment for 30 s, 72°C for 1,000 bp/min, and a final extension at 72°C for 10 min. The annealing temperature of the polymorphism fragments of *ACSF3* were 60°C. The DNA samples of 135 Chinese Simmental were amplified by PCR. Next, PCR amplification was performed with the primer pairs of SNPs of *ACSF3* as per Table 1. The specificity of PCR products was detected by 2% agarose gel electrophoresis. Nucleotide sequences of PCR products were determined by Sanger sequencing (Sangon Biotech Shanghai, China). Briefly, the nucleotide sequence of the amplified product was determined by double-stranded sequencing using the Sanger di-deoxy chain termination method. Finally, the genotypes of the cattle at each SNP were verified by the sequencing results. Haplotype analysis of the SNPs was performed using Haploview v.4.2 (https://www.broadinstitute.org/haploview/haploview).

### Statistical Analysis

Some relevant data relating to SNPs on the *ACSF3* were calculated according to the genotyping results, which were the genotypic frequency, Hardy–Weinberg test, linkage disequilibrium analysis, and polymorphism information content. The genotype frequencies and allele frequencies were calculated for the examined Chinese Simmental-cross steers and were analyzed by the significance test. An analysis of the genotypic effects of the *ACSF3* was carried out using the GLM procedure of SPSS 13.0 for Windows.


$$\begin{array}{l} Y_{ijk}=u+{\text{y}}_{si}+{\text{m}}_{j}+{\text{e}}_{ijk} \end{array}$$

where Y_ijk_ was the phenotypic observation of the kth individual from the Simmental breed of genotype *j* in the ith-year season, u was the population mean, y_si_ was the year effect of the ith-year season, m_j_ was the genotype effect of the genotype *j*, and e_ijk_ was the random residual effect corresponding to the observed value (10).

## Results

### *ACSF3* Gene Structure Prediction and Protein Interaction Prediction in Lipid Metabolism Pathways

**Figure 1:**
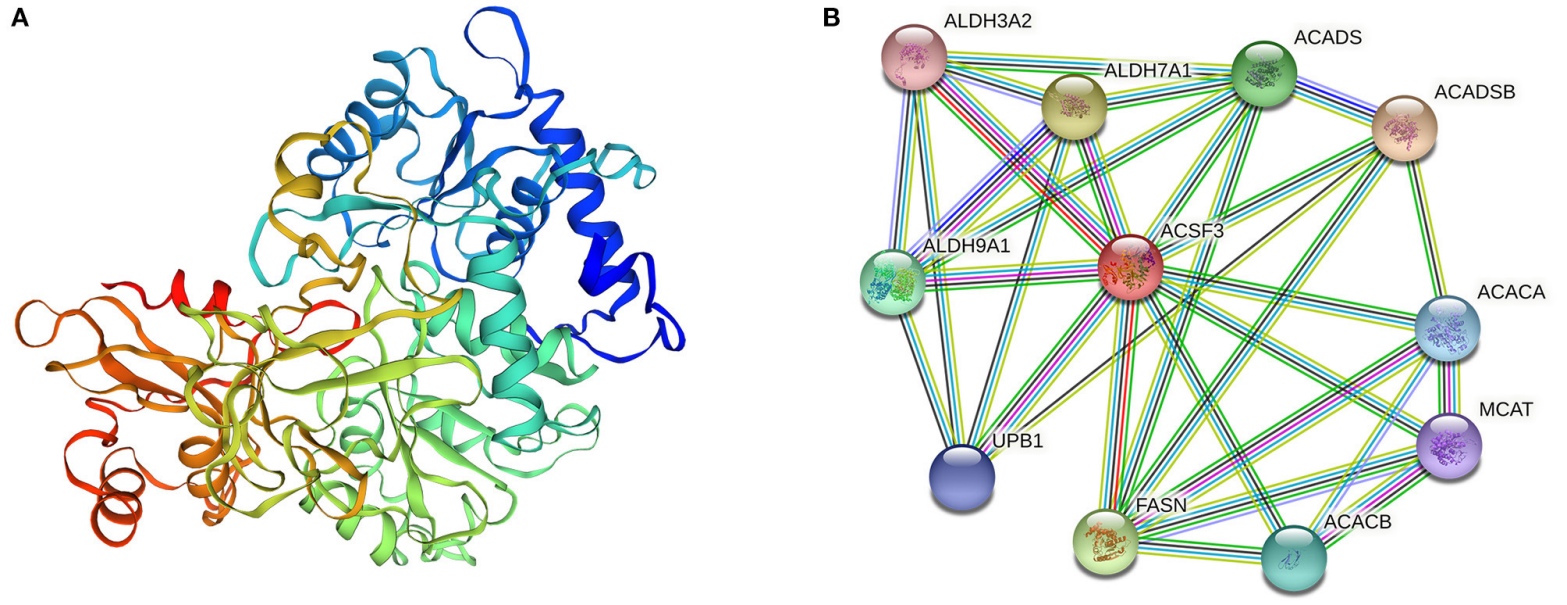
*ACSF3* gene structure prediction and protein interaction prediction in lipid metabolism pathways (14, 15). **(A)** Tertiary structure of ACSF3 protein (prediction). **(B)** Interacting proteins for *ACSF3* gene in lipid metabolism pathway of cells.

SWISS-MODEL (https://swissmodel.expasy.org/) predicted the tertiary structure of the ACSF3 protein (Figure 1A) (14). Protein interaction analysis was performed with STRING (https://cn.string-db.org/) (15). Bioinformatic data showed that 10 proteins, such as FASN, ACADSB, ACADS, ACACA, and ACACB, can interact with the ACSF3 protein (Figure 1B). The fatty acid biosynthesis and metabolism process GO terms in the biological process were enriched, implying that ACSF3 may be involved in the fatty acid biosynthetic process (GO:0006633) and the fatty acid metabolic process (GO:0006631).

### The Effect of *ACSF3* Gene Expression Level on Triglyceride Content in Cells

**Figure 2:**
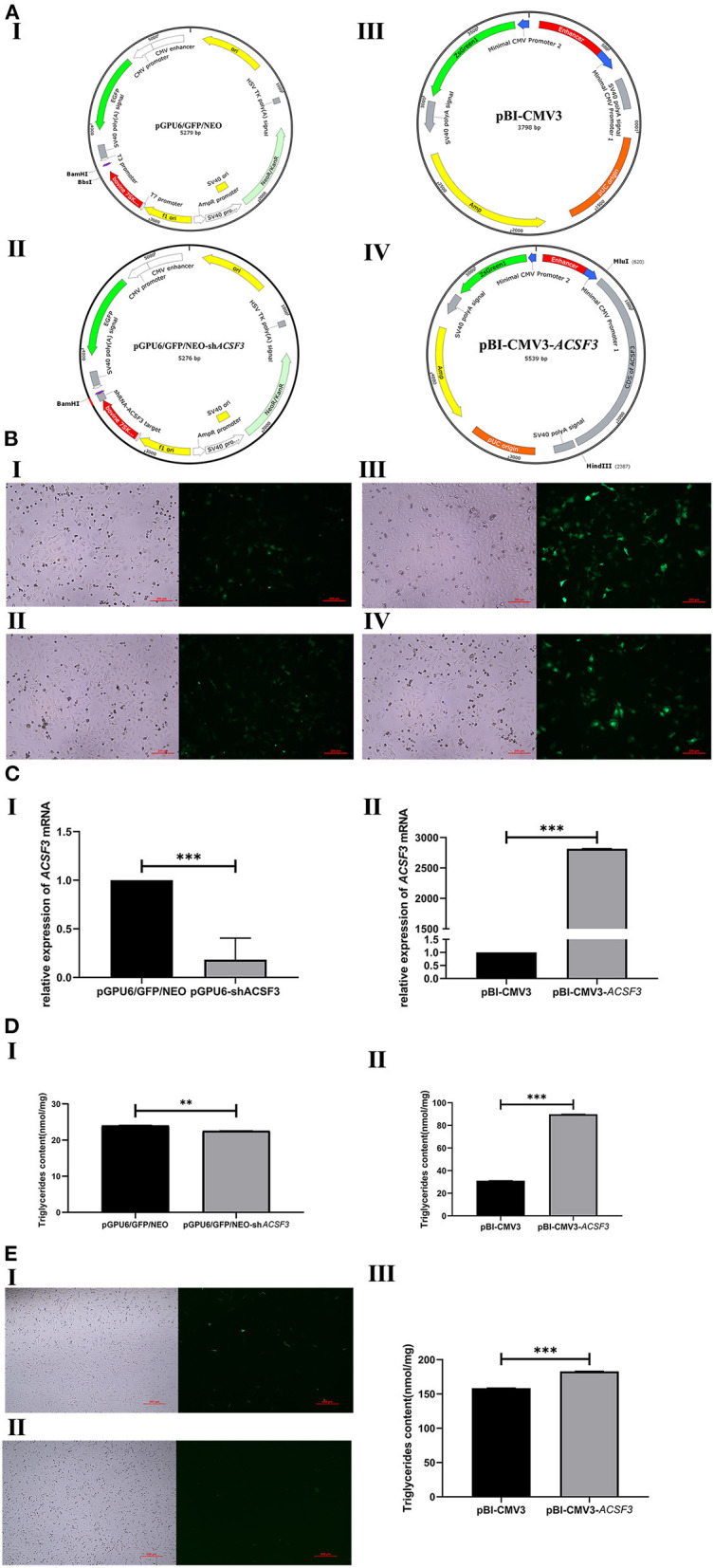
Effect of *ACSF3* gene interference or overexpression on triglyceride synthesis of transfected cells. **(A)** The maps of vectors used in this study including size. I: Control vector for RNA interference of *ACSF3* gene (pGPU6/GFP/NEO); II: RNA interference vector of *ACSF3* gene (pGPU6/GFP/NEO-sh*ACSF3*); III: Control for *ACSF3* gene over-expression vector (pBI-CMV3); IV: *ACSF3* gene over-expression vector (pBI-CMV3-*ACSF3*). **(B)** Expression of green fluorescence protein was observed in two vector groups under fluorescent microscopy. I: Cells transfected with pGPU6/GFP/Neo vectors; II: Cells transfected with pGPU6/GFP/NEO-sh*ACSF3* vectors; III: Cells transfected with pBI-CMV3 vectors; IV: Cells transfected with pBI-CMV3-*ACSF3* vectors. **(C)** The relative mRNA expression level of *ACSF3* mRNA in the two groups transfected with BMECs. I: Cells transfected with pGPU6/GFP/Neo or pGPU6/GFP/NEO-sh*ACSF3* vectors; II: Cells transfected with pBI-CMV3 or pBI-CMV3-*ACSF3* vectors. **(D)** The two vector groups caused changes in triglyceride content after transfection of BMECs. I: Cells transfected with pGPU6/GFP/NEO-sh*ACSF3* vector caused a decrease in triglyceride content; II: Cells transfected with pBI-CMV3-*ACSF3* vector caused an increase in triglyceride content. **(E)** Overexpression vectors transfected with BFFs caused changes in triglyceride content. I: Cells transfected with pBI-CMV3 vectors; II: Cells transfected with pBI-CMV3-*ACSF3* vectors. III: Cells transfected with pBI-CMV3-*ACSF3* vector caused an increase in triglyceride content. *** means *p* < 0.001, ** means *p* < 0.01.

According to the sequencing results, the siRNA target oligonucleotide sequences for *ACSF3* were successfully cloned and framed into the *BamH*I/*Bbs*I sites of the pGPU6/GFP/NEO vector. Meanwhile, the CDS fragments of *ACSF3* were cloned into the multiple cloning site (*Mlu*I/*Hind*III) of pBI-CMV3 plasmid. The green fluorescence of the transfected cells was observed in each group under fluorescent microscopy after 24 h of transfection (Figure 2), indicating that plasmids were successfully transfected into BMECs.

To investigate the effect of the pGPU6/GFP/NEO-sh*ACSF3* and pBI-CMV3-*ACSF3* vectors on the bovine *ACSF3* expression level, the relative mRNA expression level of *ACSF3* was analyzed by qRT-PCR. Compared with the control group, the mRNA expression of *ACSF3* in the pGPU6/GFP/NEO-sh*ACSF3* group was significantly reduced (*p* < 0.01, Figures 2C-I). Moreover, the expression level of *ACSF3* mRNA in the pBI-CMV3-*ACSF3* group was significantly increased compared with the control group (*p* < 0.01, Figures 2C-II).

The triglyceride contents of BMECs after transfection with the pGPU6/GFP/NEO-sh*ACSF3* and pBI-CMV3-*ACSF3* vectors were investigated. The results demonstrated that, compared with the control group, the content of TGs in the pGPU6/GFP/NEO-sh*ACSF3* group had a decreasing trend (Figures 2D-I). In addition, the content of TGs in the pBI-CMV3-*ACSF3* group increased significantly (*p* < 0.01, Figures 2D-II). Similarly, the results of *ACSF3* on TG content in BFFs are consistent with our validation on BMECs, where an over-expression of the *ACSF3* elevated triglyceride content in both types of cells (*p* < 0.01, Figures 2E-III). These results indicated that *ACSF3* could promote the synthesis of triglyceride in the lipid metabolism pathway.

### The Effect of *ACSF3* Gene Expression on Lipid Metabolism-Related Genes

**Figure 3:**
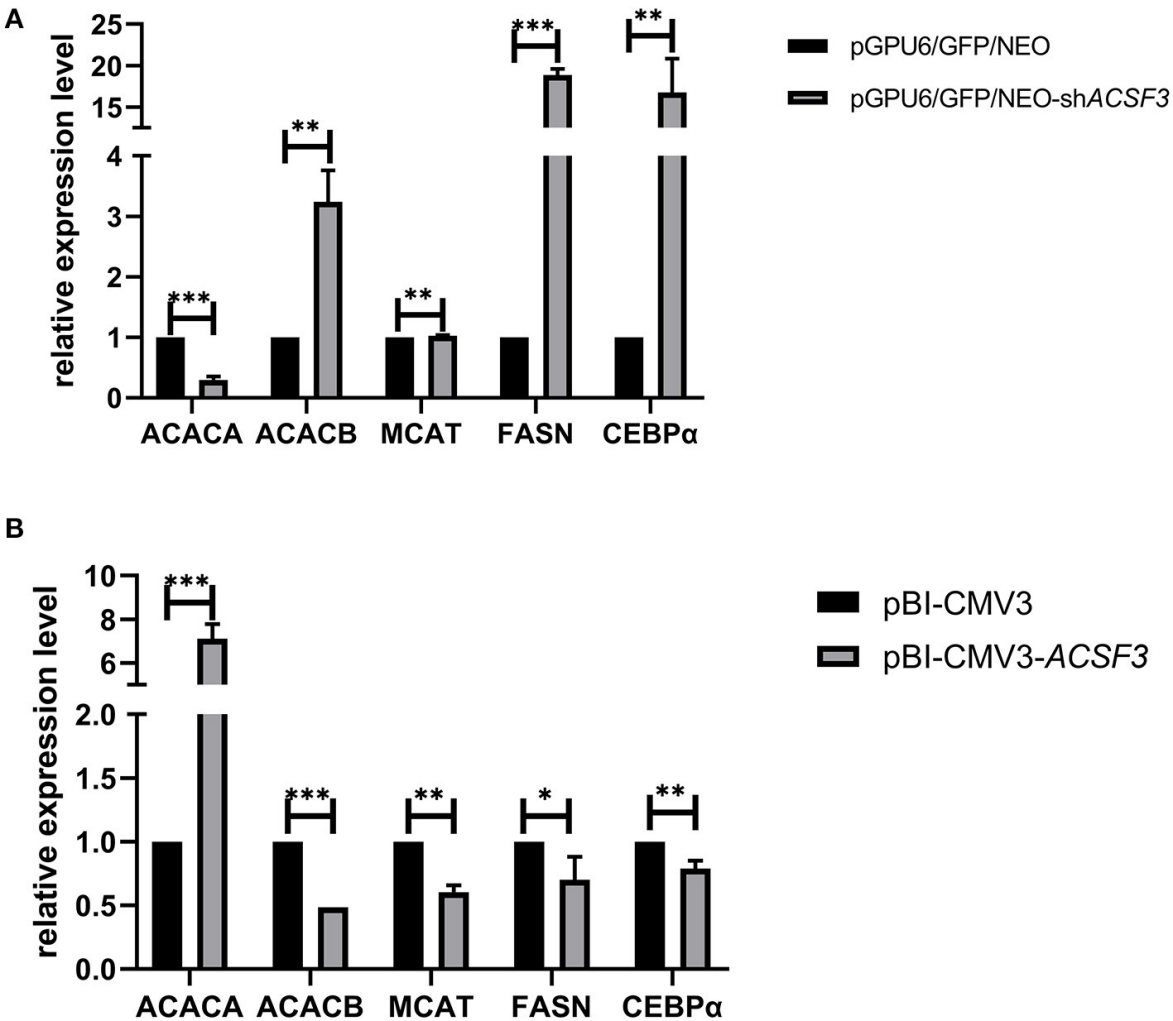
The mRNA expression of lipid metabolism-related genes in BMECs after interference **(A)** or overexpression of **(B)** the *ACSF3* gene. *** means *p* < 0.001, ** means *p* < 0.01, * means *p* < 0.05.

To further investigate the regulation of *ACSF3* on BMECs lipid metabolism-related genes, the expression levels of the related genes that interacted with the *ACSF3* gene in the lipid metabolism pathway were analyzed. The mRNA expression level of the CCAAT/enhancer binding proteinα (*CEBP*α), fatty acid synthase (*FASN*), and acetyl-CoA carboxylase beta (*ACACB*) gene in the pGPU6/GFP/NEO-sh*ACSF3* group was significantly increased compared with the control group (*p* < 0.01, Figure 3A). The mRNA expression level of the malonyl-CoA-acyl carrier protein trans acylase (*MCAT*) gene in the pGPU6/GFP/NEO-sh*ACSF3* group increased. Nevertheless, the mRNA levels of the acetyl-CoA carboxylase alpha (*ACACA*) gene of the pGPU6/GFP/NEO-sh*ACSF3* group were significantly lower than the control group (*p* < 0.01). In addition, in comparison with the control group, the mRNA expression levels of the above genes in the pBI-CMV3-*ACSF3* group exhibited an opposite trend (Figure 3B).

### Genetic Diversity of the Functional Domain of *ACSF3* Gene in Chinese Simmental Cattle

**Figure 4:**
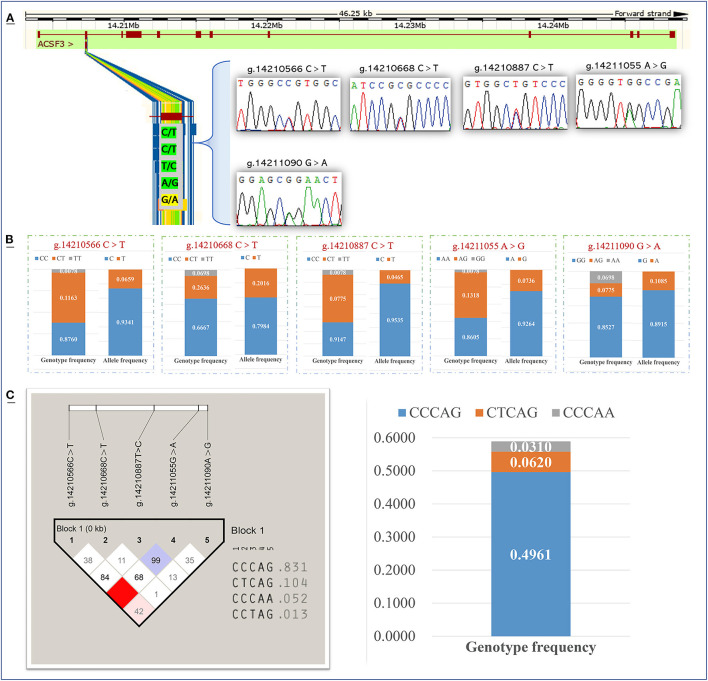
Detection and genotyping of Single-nucleotide polymorphism sites of the *ACSF3* gene in Chinese Simmental cattle. **(A)** Identify five SNPs in the second exon of the *ACSF3* gene. **(B)** Gene frequency and genotype frequency of five SNPs of *ACSF3* gene. **(C)** The haplotypes composed of five SNPs of *ACSF3* gene and the frequency analysis of haplotypes.

Meanwhile, we screened for SNPs in the key functional domain of the bovine *ACSF3* in the Chinese Simmental cattle population. According to the PCR product sequencing results, there were five polymorphisms (g.14210566 C > T, g.14210668 C > T, g.14210887 T > C, g.14211055 G > A, and g.14211090 A > G) screened in the second exon of the *ACSF3* gene in Chinese Simmental cattle (Figure 4A). Among the five loci screened, the g.14211090 A > G locus was a missense mutation (arginine to glutamine), while the other four sites were synonymous mutations. The allele frequency and genotype frequency of five *ACSF3* SNPs in Chinese Simmental cattle are presented in Figure 4. Allele C had frequencies of 0.934, 0.798, and 0.954 at the g.14210566 C > T, g.14210668 C > T, and g.14210887 T > C polymorphism sites. Allele A had a frequency of 0.926 at the g.14211055 G > A polymorphism site. Allele G had a frequency of 0.892 at the g.14211090 A > G polymorphism site (Figure 4B).

A haplotype analysis was performed on five SNPs loci in this population. Among the different genotypes, the frequency of the three haplotypes (CCCAG, CTCAG, and CCCAA) was higher than 0.03, and the frequency of the haplotype CCCAG was 0.496, which was the major haplotype (Figure 4C).

### Association Analysis of *ACSF3* Gene SNP With Economic Traits and Fatty Acids in Chinese Simmental Cattle

Regarding economic traits, the association analysis results revealed that, at the g.14211090 G > A locus, for the individuals with the G allele homozygous, a higher value of the eye muscle area, spleen weight, and oxtail weight (71.33 ± 8.80 cm^2^, 0.75 ± 0.14 kg, and 1.17 ± 0.19 kg) were observed compared with the heterozygous (65.20 ± 5.90 cm^2^, 0.65 ± 0.10 kg, and 1.03 ± 0.17 kg) individuals (*p* = 0.038, 0.040, 0.037, Table 2). Furthermore, the individuals with the TT genotype (5.99 ± 0.29) at g.14210668 C > T had a significantly higher pH (24h) than individuals with the CT genotype (5.80 ± 0.17, *p* = 0.008). The values of the dressing percentage in individuals of the TT genotype (51.82 ± 1.70%) were also significantly higher than in that of the CT genotype (50.13 ± 2.42%, *p* = 0.038). The g.14210566 C > T and g.14211055 A > G loci showed a strong linkage relationship (*r*^2^ = 0.887, LOD = 19.92).

**Table 2:** Association analysis of five SNPs in the second exon of *ACSF3* gene and economic traits in Chinese Simmental Cattle.

**Traits**	**g.14210566 C>T**		**g.14210668 C>T**			**g.14210887 C>T**		**g.14211055 A>G**		**g.14211090 G>A**		
	**CC (113)**	**CT (15)**	**CC (86)**	**CT (34)**	**TT (9)**	**CC (118)**	**TC (10)**	**AA (111)**	**GA (17)**	**AA (9)**	**AG (10)**	**GG (110)**
LW (kg)	460.77 ± 55.53^a^	429.47 ± 59.05^b^	457.94 ± 53.00	458.65 ± 65.46	441.00 ± 55.83	454.84 ± 56.03	464.40 ± 31.88	461.26 ± 55.87^a^	430.00 ± 55.57^b^	472.67 ± 86.53	432.25 ± 56.52	457.90 ± 53.38
CW (kg)	234.02 ± 32.07^a^	214.63 ± 34.54^b^	232.67 ± 31.29	230.09 ± 36.42	229.18 ± 34.69	230.91 ± 32.84	232.70 ± 16.94	234.24 ± 32.24^a^	215.48 ± 32.88^b^	234.02 ± 48.14	214.28 ± 35.29	233.15 ± 30.86
DP (%)	50.73 ± 2.13	49.87 ± 2.35	50.73 ± 2.07	50.13 ± 2.42^b^	51.82 ± 1.70^a^	50.69 ± 2.18	50.14 ± 2.22	50.73 ± 2.13	50.01 ± 2.32	49.48 ± 3.29	49.42 ± 2.80	50.86 ± 1.94
BW (kg)	21.19 ± 3.03	19.37 ± 2.39	21.10 ± 3.00	20.70 ± 2.90	21.04 ± 3.72	20.84 ± 2.98	22.20 ± 2.79	21.32 ± 3.04	19.29 ± 2.25	21.62 ± 3.37	19.59 ± 2.67	21.06 ± 2.99
FW (kg)	23.65 ± 2.67	22.61 ± 3.21	23.61 ± 2.52	23.61 ± 3.13	22.50 ± 3.15	23.47 ± 2.76	23.53 ± 1.19	23.68 ± 2.68	22.56 ± 3.00	23.60 ± 3.86	21.98 ± 2.67	23.67 ± 2.62
FHW (kg)	5.71 ± 0.58	5.39 ± 0.70	5.68 ± 0.56	5.68 ± 0.70	5.61 ± 0.64	5.66 ± 0.61	5.90 ± 0.47	5.71 ± 0.58	5.40 ± 0.66	5.76 ± 0.98	5.51 ± 0.69	5.68 ± 0.56
HHW (kg)	4.81 ± 0.56	4.61 ± 0.48	4.76 ± 0.57	4.83 ± 0.53	4.79 ± 0.46	4.77 ± 0.55	4.95 ± 0.54	4.81 ± 0.56	4.61 ± 0.46	4.94 ± 0.73	4.55 ± 0.40	4.79 ± 0.54
TaW (kg)	48.05 ± 5.87	44.32 ± 5.18	47.48 ± 5.88	48.37 ± 6.02	46.00 ± 5.55	47.46 ± 5.81	47.55 ± 4.12	48.09 ± 5.92	44.50 ± 4.88	47.92 ± 7.61	44.40 ± 6.17	47.88 ± 5.67
RRAW (kg)	8.24 ± 0.91	8.04 ± 0.73	8.20 ± 0.83	8.36 ± 0.99	7.71 ± 1.02	8.14 ± 0.86	8.76 ± 0.87	8.26 ± 0.91	7.96 ± 0.72	8.73 ± 1.23	8.03 ± 0.92	8.18 ± 0.86
OmW (kg)	4.57 ± 0.67	4.39 ± 0.71	4.54 ± 0.65	4.50 ± 0.72	4.68 ± 0.75	4.55 ± 0.69	4.55 ± 0.47	4.58 ± 0.67	4.32 ± 0.70	4.93 ± 0.90	4.48 ± 0.74	4.52 ± 0.65
HW (kg)	1.51 ± 0.20	1.38 ± 0.19	1.49 ± 0.21	1.50 ± 0.21	1.47 ± 0.17	1.48 ± 0.20	1.57 ± 0.14	1.51 ± 0.20	1.38 ± 0.18	1.53 ± 0.28	1.39 ± 0.23	1.50 ± 0.19
LW (kg)	4.74 ± 0.65	4.56 ± 0.67	4.72 ± 0.64	4.77 ± 0.70	4.45 ± 0.59	4.72 ± 0.64	4.55 ± 0.70	4.74 ± 0.66	4.57 ± 0.64	4.74 ± 0.87	4.55 ± 0.40	4.72 ± 0.66
LTW (kg)	2.78 ± 0.37	2.53 ± 0.26	2.71 ± 0.36	2.81 ± 0.39	2.90 ± 0.33	2.74 ± 0.37	2.81 ± 0.38	2.79 ± 0.37	2.52 ± 0.25	2.71 ± 0.55	2.59 ± 0.27	2.77 ± 0.36
KW (kg)	1.00 ± 0.13	0.92 ± 0.15	0.98 ± 0.12	1.02 ± 0.15	1.00 ± 0.16	0.99 ± 0.14	0.97 ± 0.09	1.01 ± 0.13	0.91 ± 0.14	1.05 ± 0.12	0.95 ± 0.11	0.99 ± 0.14
RAW (kg)	1.59 ± 0.54	1.54 ± 0.63	1.59 ± 0.57	1.56 ± 0.53	1.60 ± 0.49	1.58 ± 0.54	1.40 ± 0.40	1.60 ± 0.54	1.50 ± 0.59	1.53 ± 0.39	1.50 ± 0.65	1.59 ± 0.55
CPW (kg)	0.53 ± 0.07	0.49 ± 0.07	0.53 ± 0.07	0.51 ± 0.07	0.53 ± 0.05	0.52 ± 0.07	0.55 ± 0.06	0.53 ± 0.07	0.50 ± 0.07	0.50 ± 0.05	0.51 ± 0.09	0.53 ± 0.07
TeW (kg)	0.61 ± 0.12	0.56 ± 0.13	0.62 ± 0.12	0.60 ± 0.13	0.55 ± 0.09	0.61 ± 0.12	055 ± 0.11	0.62 ± 0.12	0.55 ± 0.13	0.51 ± 0.10^b^	0.60 ± 0.14	0.62 ± 0.12^a^
GFW (kg)	1.10 ± 0.25	0.98 ± 0.36	1.07 ± 0.26	1.17 ± 0.30	1.06 ± 0.25	1.09 ± 0.27	1.05 ± 0.24	1.10 ± 0.25	1.00 ± 0.35	1.17 ± 0.31	0.96 ± 0.39	1.10 ± 0.25
SW (kg)	0.75 ± 0.15	0.67 ± 0.12	0.74 ± 0.14	0.75 ± 0.15	0.73 ± 0.15	0.74 ± 0.15	0.72 ± 0.09	0.75 ± 0.15	0.69 ± 0.12	0.76 ± 0.22	0.65 ± 0.10^b^	0.75 ± 0.14^a^
OxW (kg)	1.17 ± 0.19	1.07 ± 0.22	1.18 ± 0.20	1.13 ± 0.19	1.05 ± 0.17	1.15 ± 0.19	1.21 ± 0.16	1.17 ± 0.19	1.07 ± 0.21	1.15 ± 0.22	1.03 ± 0.17^b^	1.17 ± 0.19^a^
pH (0 h)	6.81 ± 0.25	6.84 ± 0.18	6.83 ± 0.26	6.80 ± 0.21	6.73 ± 0.21	6.81 ± 0.25	6.82 ± 0.22	6.81 ± 0.25	6.82 ± 0.18	6.71 ± 0.17	6.84 ± 0.23	6.82 ± 0.25
pH (24 h)	5.85 ± 0.19	5.90 ± 0.20	5.87 ± 0.18	5.80 ± 0.17^B^	5.99 ± 0.29^A^	5.86 ± 0.19	5.80 ± 0.13	5.85 ± 0.19	5.88 ± 0.20	5.93 ± 0.16	5.92 ± 0.20	5.85 ± 0.19
CL (cm)	146.50 ± 5.84	142.47 ± 6.12	145.98 ± 5.81	146.38 ± 6.35	144.56 ± 6.78	145.87 ± 6.03	146.10 ± 4.53	146.55 ± 5.85	142.59 ± 5.95	147.44 ± 6.29	143.20 ± 6.20	146.12 ± 5.93
CD (cm)	65.54 ± 3.04	64.93 ± 4.23	65.44 ± 3.02	65.68 ± 3.67	65.33 ± 3.00	65.48 ± 3.24	65.50 ± 2.59	65.55 ± 3.03	64.94 ± 4.10	67.78 ± 3.46^a^	65.40 ± 4.58	65.31 ± 2.97^b^
CBD (cm)	66.53 ± 3.60	65.87 ± 3.60	66.64 ± 3.46	66.09 ± 3.93	66.44 ± 3.81	66.44 ± 3.67	66.35 ± 2.33	66.54 ± 3.62	65.88 ± 3.44	66.94 ± 3.71	66.00 ± 3.77	66.49 ± 3.60
HLC (cm)	48.20 ± 3.97	47.00 ± 4.39	48.49 ± 3.99	46.93 ± 3.95	48.53 ± 4.08	48.03 ± 4.06	48.53 ± 3.81	48.19 ± 4.00	47.18 ± 4.17	46.94 ± 3.43	47.85 ± 4.61	48.20 ± 4.02
HLW (cm)	44.68 ± 2.62	44.13 ± 3.20	44.74 ± 2.65	44.00 ± 2.87	45.61 ± 1.82	44.56 ± 2.77	45.10 ± 1.56	44.70 ± 2.64	44.06 ± 3.00	44.56 ± 3.39	44.85 ± 3.16	44.59 ± 2.60
HLL (cm)	84.39 ± 2.76	84.37 ± 2.58	84.43 ± 2.79	84.54 ± 2.53	83.48 ± 2.85	84.31 ± 2.75	85.40 ± 2.38	84.41 ± 2.87	84.24 ± 2.46	84.11 ± 2.76	83.90 ± 2.51	84.46 ± 2.75
TMT (cm)	17.44 ± 1.54	17.41 ± 1.57	17.47 ± 1.64	17.42 ± 1.45	17.42 ± 1.01	17.49 ± 1.53	17.11 ± 1.80	17.44 ± 1.55	17.36 ± 1.51	17.20 ± 1.56	17.92 ± 1.53	17.43 ± 1.55
WMT (cm)	6.02 ± 0.44	5.81 ± 0.39	6.01 ± 0.43	5.96 ± 0.48	5.93 ± 0.33	5.99 ± 0.43	6.00 ± 0.42	6.02 ± 0.44	5.81 ± 0.37	6.04 ± 0.55	5.81 ± 0.45	6.00 ± 0.42
BFT (cm)	0.27 ± 0.11	0.27 ± 0.12	0.28 ± 0.11	0.26 ± 0.12	0.26 ± 0.10	0.28 ± 0.12	0.21 ± 0.07	0.27 ± 0.11	0.28 ± 0.13	0.29 ± 0.15	0.29 ± 0.10	0.27 ± 0.12
FCR (%)	22.24 ± 8.05	22.20 ± 7.48	22.66 ± 7.99	20.74 ± 7.76	24.44 ± 8.08	22.34 ± 8.00	20.80 ± 7.44	22.19 ± 8.08	22.53 ± 7.27	21.67 ± 5.66	21.10 ± 8.86	22.44 ± 8.06
MS	5.88 ± 0.35	5.87 ± 0.35	5.90 ± 0.34	5.82 ± 0.39	6.00 ± 0.00	5.88 ± 0.35	6.00 ± 0.00	5.88 ± 0.35	5.88 ± 0.33	5.89 ± 0.33	5.90 ± 0.32	5.88 ± 0.35
EMA (cm^2^)	71.82 ± 9.04^a^	64.53 ± 5.38^b^	71.62 ± 8.71	69.15 ± 9.58	72.11 ± 8.87	70.86 ± 8.87	72.10 ± 10.71	71.99 ± 9.02^A^	64.29 ± 5.16^B^	73.44 ± 11.84^a^	65.20 ± 5.90^ab^	71.33 ± 8.80^b^
MC	4.95 ± 0.83	4.93 ± 0.88	4.99 ± 0.85	4.76 ± 0.78	5.33 ± 0.87	4.97 ± 0.86	4.90 ± 0.57	4.96 ± 0.83	4.82 ± 0.88	4.89 ± 0.60	4.90 ± 0.88	4.96 ± 0.86
FCS	3.59 ± 0.62^a^	3.13 ± 0.64^b^	3.47 ± 0.65	3.59 ± 0.56	3.89 ± 0.93	3.53 ± 0.66	3.50 ± 0.53	3.59 ± 0.62^a^	3.18 ± 0.64^b^	3.56 ± 0.73	3.20 ± 0.92	3.55 ± 0.61

a, b *Means with different letters were significant difference (p < 0.05)*,

A, B *Means with different letters were significant difference (p < 0.01). SD, standard deviation; LW, liveweight; CW, carcass weight; DP, dressing percentage; BW, bone weight; FW, front weight; FHW, front hoof weight; HHW, hind hoof weight; TaW, tare weight; RRAW, rumen, reticulum and abomasum weight; OmW, omasum weight; HW, heart weight; LW, liver weight; LTW, lung and trachea weight; KW, kidney weight; RAW, renal adipose weight; CPW, cow penis weight; TeW, testicular weight; GFW, genital fat weight; SW, spleen weight; OxW, oxtail weight; CL, carcass length; CD, carcass depth; CBD, carcass breast depth; HLC, hind legs circumference; HLW, hind legs width; HLL, hind legs length; TMT, thigh meat thickness; WMT, waist meat thickness; BFT, back-fat thickness; FCR, carcass fat coverage rate; MS, marbling score; EMA, eye muscle area; MC, meat color; FCS, fat color score*.

Regarding fatty acid composition, as shown in Table 3, the linoleic acid content of the AG genotype (0.131 ± 0.056 g/100g) at the g.14211090 G > A locus was significantly higher than that of the GG genotype (0.101 ± 0.032 g/100g, *p* = 0.008, Table 3). The individuals with the TT genotype (0.130 ± 0.054 g/100g) at the g.14210668 C > T locus had higher linoleic acid than the other two genotypes (0.102 ± 0.035 g/100 g, 0.100 ± 0.029 g/100g, *p* = 0.023, *p* = 0.022). Similarly, individuals with the TC genotype (0.129 ± 0.054 g/100g) at the g.14210887 C > T locus had higher levels of linoleic acid than the other genotype (0.102 ± 0.032 g/100g, *p* = 0.017).

**Table 3:** Association analysis of five SNPs in the second exon of *ACSF3* gene and fatty acids in Chinese Simmental Cattle.

**Types of fatty acids (g/100g)**	**g.14210566 C>T**		**g.14210668 C>T**			**g.14210887 C>T**		**g.14211055 A>G**		**g.14211090 G>A**		
	**CC (113)**	**CT (15)**	**CC (86)**	**CT (34)**	**TT (9)**	**CC (118)**	**TC (10)**	**AA (111)**	**GA (17)**	**AA (9)**	**AG (10)**	**GG (110)**
Myristic acid	0.020 ± 0.017	0.024 ± 0.018	0.020 ± 0.017	0.021 ± 0.019	0.023 ± 0.013	0.020 ± 0.017	0.025 ± 0.019	0.020 ± 0.017	0.025 ± 0.017	0.025 ± 0.022	0.029 ± 0.021	0.020 ± 0.016
Myristoleic acid	0.002 ± 0.005	0.003 ± 0.004	0.002 ± 0.005	0.003 ± 0.004	0.002 ± 0.002	0.002 ± 0.005	0.004 ± 0.005	0.002 ± 0.005	0.002 ± 0.004	0.003 ± 0.005	0.003 ± 0.004	0.002 ± 0.005
Palmitic acid	0.258 ± 0.191	0.300 ± 0.184	0.265 ± 0.198	0.248 ± 0.181	0.296 ± 0.149	0.257 ± 0.190	0.313 ± 0.189	0.254 ± 0.191	0.318 ± 0.180	0.306 ± 0.211	0.347 ± 0.210	0.251 ± 0.185
Palmitoleic acid	0.027 ± 0.031	0.031 ± 0.021	0.028 ± 0.034	0.026 ± 0.020	0.024 ± 0.013	0.027 ± 0.030	0.032 ± 0.023	0.027 ± 0.031	0.031 ± 0.020	0.029 ± 0.021	0.037 ± 0.023	0.027 ± 0.031
Margaric acid	0.011 ± 0.007	0.013 ± 0.007	0.011 ± 0.007	0.012 ± 0.008	0.014 ± 0.007	0.011 ± 0.007	0.014 ± 0.008	0.011 ± 0.007	0.014 ± 0.007	0.015 ± 0.010	0.015 ± 0.009	0.011 ± 0.007
Heptadecenoic acid	0.005 ± 0.007	0.007 ± 0.005	0.006 ± 0.007	0.005 ± 0.006	0.003 ± 0.005	0.005 ± 0.007	0.005 ± 0.006	0.005 ± 0.007	0.007 ± 0.005	0.005 ± 0.006	0.009 ± 0.005	0.005 ± 0.007
Stearic acid	0.187 ± 0.112	0.221 ± 0.111	0.187 ± 0.104	0.188 ± 0.126	0.235 ± 0.131	0.186 ± 0.110	0.238 ± 0.140	0.184 ± 0.110	0.240 ± 0.117	0.241 ± 0.178	0.250 ± 0.123	0.181 ± 0.103
Oleic acid	0.365 ± 0.378	0.394 ± 0.224	0.379 ± 0.414	0.329 ± 0.230	0.404 ± 0.201	0.363 ± 0.372	0.424 ± 0.237	0.362 ± 0.381	0.410 ± 0.215	0.397 ± 0.271	0.462 ± 0.245	0.357 ± 0.377
Linoleic acid	0.101 ± 0.033^b^	0.120 ± 0.050^a^	0.102 ± 0.035^b^	0.100 ± 0.029^b^	0.130 ± 0.054^a^	0.102 ± 0.032^b^	0.129 ± 0.054^a^	0.101 ± 0.032^b^	0.122 ± 0.047^a^	0.106 ± 0.036	0.131 ± 0.056^A^	0.101 ± 0.032^B^
α-linolenic acid	0.006 ± 0.006	0.009 ± 0.013	0.007 ± 0.008	0.005 ± 0.005	0.010 ± 0.010	0.006 ± 0.006^B^	0.013 ± 0.015^A^	0.006 ± 0.006	0.009 ± 0.012	0.005 ± 0.005	0.011 ± 0.016^a^	0.006 ± 0.006^b^
Arachic acid	0.001 ± 0.003	0.001 ± 0.002	0.000 ± 0.001	0.001 ± 0.005	0.001 ± 0.001	0.001 ± 0.003	0.001 ± 0.002	0.001 ± 0.003	0.001 ± 0.002	0.001 ± 0.001	0.002 ± 0.002	0.001 ± 0.003
Eicosanic acid	0.001 ± 0.002	0.000 ± 0.001	0.000 ± 0.002	0.001 ± 0.002	0.000 ± 0.001	0.000 ± 0.002	0.001 ± 0.001	0.001 ± 0.002	0.000 ± 0.001	0.000 ± 0.001	0.001 ± 0.001	0.000 ± 0.002
Dihomo-γ-linolenic acid	0.010 ± 0.003	0.010 ± 0.003	0.010 ± 0.003	0.010 ± 0.003	0.009 ± 0.002	0.010 ± 0.003	0.011 ± 0.002	0.010 ± 0.003	0.010 ± 0.003	0.010 ± 0.001	0.011 ± 0.003	0.010 ± 0.003
Arachidonic acid	0.049 ± 0.013	0.055 ± 0.019	0.050 ± 0.014	0.051 ± 0.013	0.049 ± 0.011	0.050 ± 0.013	0.056 ± 0.018	0.049 ± 0.013	0.053 ± 0.018	0.051 ± 0.012	0.059 ± 0.020^a^	0.049 ± 0.013^b^

a, b *Means with different letters were significant difference (p < 0.05)*,

A, B *Means with different letters were significant difference (p < 0.01). SD, standard deviation*.

### Correlation Analysis of Polymorphic Locus Haplotypes, Economic Traits and Fatty Acids in Chinese Simmental Cattle

The haplotype CTCAG was significantly correlated with pH (24 h) and fat color score (6.03 ± 0.27, 5.87 ± 0.18, 4.00 ± 0.93, 3.51 ± 0.59, *p* = 0.022, and *p* = 0.042, Table 4), and the weight of the lung and trachea was significantly higher than in other haplotype individuals (2.90 ± 0.35 kg, 2.23 ± 0.30 kg, *p*= 0.002). The front hoof weight of the haplotype CCCAG (5.71 ± 0.53 kg) was significantly higher than that of CCCAA (5.13 ± 0.52 kg, *p* = 0.044), the weight of oxtail (1.20 ± 0.19 kg) was significantly higher than that of CTCAG (1.03 ± 0.16 kg, *p* = 0.015), and the weight of the testis (0.63 ± 0.12 kg) was significantly higher than that of CCCAA (0.46 ± 0.10 kg, *p* = 0.004). Similar to the results of the correlation analysis between SNPs and fatty acid content, the content of linoleic acid in haplotype CTCAG (0.130 ± 0.058 g/100g) was significantly higher than that of haplotype CCCAG (0.097 ± 0.028 g/100 g, *p* < 0.01, Table 5).

**Table 4:** Association analysis of three haplotypes and economic traits in Chinese Simmental Cattle.

**Types of fatty acids (g/100g)**	**Haplotype (Mean ± SD)**		
	**Hap1 CCCAA (4)**	**Hap2 CCCAG (63)**	**Hap3 CTCAG (8)**
Liveweight (kg)	423.63 ± 21.73	461.65 ± 50.83	442.94 ± 59.36
Carcass weight (kg)	213.00 ± 18.72	235.35 ± 30.33	229.88 ± 37.02
Dressing percentage	50.22 ± 2.00	50.91 ± 2.01	51.73 ± 1.80
Bone weight (kg)	18.68 ± 0.84	21.26 ± 3.04	20.88 ± 3.94
Front weight (kg)	21.44 ± 1.56	23.90 ± 2.52	22.63 ± 3.35
Front hoof weight (kg)	5.13 ± 0.52^b^	5.71 ± 0.53^a^	5.59 ± 0.68
Hind hoof weight (kg)	4.38 ± 0.48	4.82 ± 0.59	4.83 ± 0.47
Tare weight (kg)	43.20 ± 6.94	48.13 ± 5.59	46.55 ± 5.67
Rumen, reticulum and abomasum weight (kg)	7.79 ± 0.18	8.25 ± 0.83	7.81 ± 1.05
Omasum weight (kg)	4.75 ± 0.35	4.59 ± 0.66	4.79 ± 0.73
Heart weight (kg)	1.35 ± 0.09	1.50 ± 0.20	1.47 ± 0.18
Liver weight (kg)	4.19 ± 0.41	4.78 ± 0.62	4.48 ± 0.62
Lung and trachea weight (kg)	2.23 ± 0.30^AB^	2.79 ± 0.35^B^	2.90 ± 0.35^A^
Kidney weight (kg)	0.98 ± 0.04	1.00 ± 0.13	1.02 ± 0.16
Renal adipose weight (kg)	1.45 ± 0.20	1.63 ± 0.57	1.70 ± 0.40
Cow penis weight (kg)	0.48 ± 0.04	0.53 ± 0.07	0.52 ± 0.05
Testicular weight (kg)	0.46 ± 0.10^B^	0.63 ± 0.12^A^	0.56 ± 0.09
Genital fat weight (kg)	1.07 ± 0.33	1.10 ± 0.22	1.07 ± 0.27
Spleen weight (kg)	0.63 ± 0.09	0.76 ± 0.14	0.73 ± 0.16
Oxtail weight (kg)	1.05 ± 0.12	1.20 ± 0.19^a^	1.03 ± 0.16^b^
pH(0 h)	6.64 ± 0.18	6.84 ± 0.27	6.75 ± 0.22
pH(24 h)	5.95 ± 0.15	5.87 ± 0.18^b^	6.03 ± 0.27^a^
Carcass length (cm)	143.00 ± 3.74	146.70 ± 5.71	144.25 ± 7.19
Carcass depth (cm)	66.00 ± 2.31	65.48 ± 3.01	65.25 ± 3.20
Carcass breast depth (cm)	64.88 ± 0.85	66.78 ± 3.69	66.25 ± 4.03
Hind legs circumference (cm)	44.75 ± 1.50	48.66 ± 4.11	47.50 ± 2.83
Hind legs width (cm)	43.25 ± 3.20	44.76 ± 2.63	45.50 ± 1.91
Hind legs length (cm)	82.13 ± 2.46	84.61 ± 2.89	83.54 ± 3.04
Thigh meat thickness (cm)	16.80 ± 0.89	17.54 ± 1.64	17.31 ± 1.02
Waist meat thickness (cm)	5.75 ± 0.50	6.04 ± 0.44	5.95 ± 0.35
Back-fat thickness (cm)	0.28 ± 0.22	0.28 ± 0.11	0.26 ± 0.11
Carcass fat coverage rate (%)	21.50 ± 2.38	22.70 ± 8.09	25.63 ± 7.76
Marbling score	5.75 ± 0.50	5.92 ± 0.33	6.00 ± 0.00
Eye muscle area (cm^2^)	67.00 ± 9.63	73.14 ± 8.57	71.25 ± 9.07
Meat color	4.50 ± 0.58	5.11 ± 0.86	5.38 ± 0.92
Fat color score	3.50 ± 0.58	3.51 ± 0.59^b^	4.00 ± 0.93^a^

a, b *Means with different letters were significant difference (p < 0.05)*,

A, B *Means with different letters were significant difference (p < 0.01). SD, standard deviation*.

**Table 5:** Association analysis of three haplotypes and fatty acids in Chinese Simmental Cattle.

**Types of fatty acids (g/100g)**	**Haplotype (Mean ± SD)**		
	**Hap1 CCCAA**	**Hap2 CCCAG**	**Hap3 CTCAG**
	**(4)**	**(63)**	**(8)**
Myristic acid	0.013 ± 0.005	0.020 ± 0.017	0.023 ± 0.014
Myristoleic acid	0.000 ± 0.001	0.002 ± 0.006	0.002 ± 0.002
Palmitic acid	0.180 ± 0.014	0.261 ± 0.212	0.302 ± 0.159
Palmitoleic acid	0.017 ± 0.005	0.029 ± 0.039	0.024 ± 0.014
Margaric acid	0.009 ± 0.002	0.010 ± 0.007	0.015 ± 0.008
Heptadecenoic acid	0.002 ± 0.004	0.006 ± 0.008	0.003 ± 0.005
Stearic acid	0.149 ± 0.034	0.178 ± 0.102	0.243 ± 0.138
Oleic acid	0.247 ± 0.017	0.387 ± 0.475	0.409 ± 0.214
Linoleic acid	0.098 ± 0.022	0.097 ± 0.028^B^	0.130 ± 0.058^A^
α-linolenic acid	0.006 ± 0.005	0.005 ± 0.006	0.010 ± 0.011
Arachic acid	0.000 ± 0.001	0.000 ± 0.001	0.001 ± 0.002
Eicosanic acid	0.000 ± 0.001	0.000 ± 0.003	0.001 ± 0.001
Dihomo-γ-linolenic acid	0.010 ± 0.000	0.010 ± 0.003	0.009 ± 0.002
Arachidonic acid	0.053 ± 0.012	0.049 ± 0.013	0.047 ± 0.011

A, B *Means with different letters were significant difference (p < 0.01). SD, standard deviation*.

## Discussion

The candidate genes associated with carcass and meat quality traits have been extensively validated in the livestock industry in the expectation of obtaining better economic characteristics. Single nucleotide polymorphisms as DNA markers for genetic and molecular breeding preferences, combining gene function studies with association analysis is more conducive to fully explore the relationships and characteristics of genes. We performed transcriptome sequencing analysis in cattle with different fat deposition traits and obtained differentially expressed genes, including *ACSF3*. Acyl-CoA synthetase family member 3 (ACSF3), an essential enzyme that activates fatty acids through the formation of thioester bonds to form acyl-CoA, serves as the substrate for both *de novo* fatty acid synthesis and oxidation (16). *ACSF3* is valuable to be studied as a gene that plays a role in the process of lipid metabolism.

In this study, we successfully constructed the RNA interference and over-expression vectors of *ACSF3*, and found that *ACSF3* mRNA levels were positively correlated with triglyceride content in cells. Five SNP loci were screened in a population of 135 Chinese Simmental cattle, and haplotype analysis and association analysis were performed on these five SNP loci. The association between SNP loci, haplotypes and economic traits and fatty acid composition was investigated, and the results showed that these five SNP loci and haplotypes were significantly associated with economic traits and fatty acid composition of Chinese Simmental cattle.

Both milk lipid metabolism and meat lipid metabolism are of interest in livestock industry research, and in the present study, we were surprised to find that increased mRNA levels of *ACSF3* caused an increase in triglyceride content in both BMECs and BFFs. This suggested to us that *ACSF3* may play a similar role in lipid metabolism, without tissue specificity. This may be due to main role of ACSF3 is to activate the toxic, endogenous antimetabolite malonate into malonyl-CoA that can be decarboxylated to acetyl-CoA and therefore fully oxidized within the TCA cycle. And one function of *ACSF3* that has been proposed was that it generated malonyl-CoA in the matrix of the mitochondria to enable mitochondrial type II (mtFASII) fatty acid synthesis (17, 18).

We found that it was co-annotated with genes such as *FASN* and *ACACA* in the fatty acid biosynthesis and fatty acid metabolism pathway of KEGG by predicting the *ACSF3* protein interaction network. We hypothesized that *ACSF3* may affect genes related to lipid metabolism and thus affect lipid metabolism. In the mitochondria, ACSF3 links malonate to CoA, producing malonyl-CoA. In addition, when *ACACA* is inhibited, TG synthesis will also be inhibited, and at the same time, fatty acid synthesis is inhibited and fatty acid oxidation is stimulated (19). The results of our qRT-PCR showed that the mRNA expression levels of *ACACA* and *ACSF3* changed consistently. This may be due to the coordination of mammalian ACACA and ACSF3 to produce the malonyl-CoA required for mitochondrial fatty acid synthesis, indicating that ACACA also plays an essential role in acetyl-CoA sensing. In mitochondria, malonyl-CoA is the main signal of energy metabolism (20). It has been proved that *FASN* inhibition leads to a rapid increase in malonyl-CoA in cells (21). The result may be the reason why we found that *ACSF3* and *FASN* have a negative regulatory relationship at the mRNA level.

We screened five SNP loci in Chinese Simmental cattle population, where the SNP of the g.14211090 A > G loci triggered missense mutations that resulted in the conversion of the encoded amino acid from arginine to glutamine. In human metabolic diseases research, *ACSF3* as a cause of combined malonic and methylmalonic aciduria (CMAMMA) has been studied. Mutations in *ACSF3* were identified, encoding the putative methylmalonyl-CoA and malonyl-CoA synthase as the cause of CMAMMA (16). A similar study found a homozygous missense allele in the non-classical CMAMMA candidate gene *ACSF3* in patients (22). Hence, it is worth investigating whether the mutation of *ACSF3* could be used as an early molecular marker for the detection of lipid metabolism in bovine.

For the carcass traits of Chinese Simmental cattle, the g.14210566 C > T and g.14211055 A > G loci were significantly associated with the liveweight and carcass weight. These results suggest that these two SNPs may be associated with growth and development in cattle. For meat quality traits of Chinese Simmental cattle, the g.14210566 C > T, g.14211055 A > G, and g.14211090 G > A loci were found to be significantly associated with the eye muscle area. In Angus bulls, eye muscle area had positive genetic (57%) and phenotypic (56%) correlations with liveweight (23). The reason why the g.14210566 C > T and g.14211055 A > G loci were found to be associated with live weight and eye muscle area was attributed to the fact that eye muscle area is genetically associated with live weight and meat quality.

Similarly, the results of an association analysis of pork quality traits and whole genomes showed that *ACSF3* was a candidate gene related to intramuscular fat (24). We examined the fatty acid composition and content of intramuscular fat, rather than measuring the percentage of intramuscular fat. We were surprised to find that all the five SNPs obtained from the screening were significantly associated with linoleic acid. Linoleic acid is one of the essential fatty acids in the human body. It plays a meaningful role in maintaining many physiological functions of the human body, such as participating in the synthesis of phospholipids and the metabolism of other lipids, and has the effect of significantly lowering serum cholesterol. Studies have shown that *ACSF3* is significantly up-regulated in the process of alcoholic liver disease, participates in fatty acid and lipid metabolism, and accelerates liver damage (25). Therefore, the SNPs of *ACSF3* can be used as a molecular marker for breeding cattle with high linoleic acidity. The effect of bovine *ACSF3* on lipid metabolism is deserving of a follow-up in-depth study. Meanwhile, two SNPs (g.14210887 C > T and g.14211090 G > A) were significantly associated with α-linolenic acid. Linoleic acid and α-linolenic acid are both beneficial to human health as polyunsaturated fatty acids. Breeding individuals with a high polyunsaturated fatty acid content in muscle is the goal of specialized beef breed selection and high-quality beef production. As a consequence, it is of great significance to screen the genes and genetic markers that determine the polyunsaturated fatty acids and to select high-quality beef cattle through the application of molecular breeding techniques such as marker-assisted selection.

The haplotype consisting of five SNPs was also associated with linoleic acid. In an association analysis between SNPs or haplotypes and meat quality traits, only the two traits of fat color score and pH (24 h) were significantly associated. The traits that were considered to be significant in the association analysis of SNPs with traits did not exactly match the results of haplotypes, which may be due to the relatively small population of cattle collected in the current experiment.

In conclusion, the function of the *ACSF3* in bovine lipid metabolism was preliminarily analyzed and we proposed an association analysis report combining SNPs with economic traits and fatty acid composition to support molecular marker-assisted selection to predict the association analysis of SNPs with economic traits of beef cattle.

## Data Availability Statement

The original contributions presented in the study are included in the article/supplementary material, further inquiries can be directed to the corresponding author/s.

## Ethics Statement

The animal study was reviewed and approved by the Animal Protection and Use Committee of Jilin University [permit no. SYXK (Ji) pzpx20181227083].

## Author Contributions

WH and XF: conceptualization, validation, and writing—original draft. WH and XL: formal analysis. RY: funding acquisition, project administration, and writing—review & editing. WH, YL, and JL: methodology. ZZ: resources. GL: software. WH and GL: visualization. All authors contributed to the article and approved the submitted version.

## Funding

This work was supported by the National Natural Science Foundation of China (31972993), the Jilin Scientific and Technological Development Program (20180101275JC), and Graduate Innovation Fund of Jilin University (101832020CX333).

## Conflict of Interest

The authors declare that the research was conducted in the absence of any commercial or financial relationships that could be construed as a potential conflict of interest.

## Publisher's Note

All claims expressed in this article are solely those of the authors and do not necessarily represent those of their affiliated organizations, or those of the publisher, the editors and the reviewers. Any product that may be evaluated in this article, or claim that may be made by its manufacturer, is not guaranteed or endorsed by the publisher.
